# Hepatic Abscess

**Published:** 1890-03-22

**Authors:** 


### SURGERY.

### HEPATIC ABSCESS.

Surgeon-General Marston contributes to the *Lancet* "Observations on Abscess of the Liver." This complaint may arise in connection with dysentery, septic matter being conveyed direct from the ulcerated intestine to the liver; secondly, from the general contamination of the system known as pyæmia from pus in other parts than the intestines; thirdly, as a malady *sui generis*, originating in the liver without connection with dysentery or the pyæmic process. Now, we venture to observe that as regards the connection of liver abscess with dysentery, although frequently seen, it may be doubted if it is really the result of the dysentery. Long ago the late Dr. Moorhead observed that in cases of liver abscess connected apparently with dysentery, there would usually be found some antecedent hepatic derangement. As respects liver abscess occurring from pyæmia, this does not frequently result. The great majority of liver abscesses in the East occur irrespective of either dysentery or pyæmia. Surgeon-General Marston mentions that the disease is most prevalent in the coast districts of the eastern hemisphere, where considerable diurnal variations of temperature leading to chill predispose to it. In a hot dry temperature, where the products of perspiration are rapidly carried off, there is less tendency to passive and continued visceral engorgement, than in conditions of atmosphere where these are less freely carried off. When the individual lives in a kind of steam bath (as in Bombay, for instance, during some part of the year) the functions of the skin fluctuate even with small changes of temperature Too much or improper diet will doubtless predispose the liver to congestion, but chill is generally the exciting cause of a liver abscess. As Dr. Marston observes, the first stage of abscess is a granular, dark-coloured degeneration, which rapidly breaks down into pus. The metastatic form of liver abscess is the most dangerous, for it is generally multiple. But, unfortunately, we cannot always say during life what form exists. It may, however, be suspected to be multiple if in connexion with dysentery, and this would form a reason against operative proceedure, for it is little use to open one abscess if another exists. Dr. Marston states there are no groups of symptoms always connected with hepatic abscess. But he insists on careful examination from time to time, both of the liver and of the chest, and a careful comparison of the results of such examination with the present condition and past history of the patient. Sallow complexion, anxious facial expression, slow gait, the presence or absence of diarrhœa, the occurrence of rigour, temperature rise, emaciation, inability to lie on the left side, depression of spirits, and hacking cough, are all frequently associated with hepatic abscess. On the other hand, cases do occur when the patient remains in apparently good health until the abscess bursts. Jaundice is frequently absent, perhaps most frequently. The bowels may or may not be constipated. Anasarca from pressure on the vena cava may or may not occur. Swelling or bulging may be quite absent, and yet a large collection of pus may exist. Often examination of the liver affords no evidence of a deep-seated abscess. Smart percussion is more likely to excite uneasiness than more gentle digital examination, and especially percussion in the posterior region of the liver. In short the liver is so large an organ, and an abscess may be in such different positions, that a different class of symptoms must arise depending in a great measure on the position of the abscess. In a second article Surgeon-General Marston thinks that operative interference should be adopted as soon as possible. The abscess should be opened antiseptically and freely, and a drainage tube "of a calibre equal to the requirements" should be used. Contrary to what has been advanced by Mr. Godlee, regarding the danger of hæmorrhage from the liver when any of the texture of the organ is divided, Marston says, that although the hæmorrhage is sometimes considerable, he has never heard of it being fatal. Some authors have surmised injury from bile finding its way into the cavity of an abscess, but Marston says if bile finds its way into an abscess cavity it acts as an antiseptic. The author also mentions that a drainage tube between the ribs may cause ulceration of an intercostal artery, and internal hæmorrhage into the sac. Most authors mention erosion of the ribs from pressure of a hard tube, but few refer to the danger of hæmorrhage from an artery of the ribs. Marston also mentions that the possibility of secondary hæmorrhage into the abscess cavity should not be lost sight of, to which again few authors refer.

